# Plasma Drug Concentration of Propranolol and Genetic Study in Chinese Han Patients With Infantile Haemangioma

**DOI:** 10.3389/fped.2022.849496

**Published:** 2022-05-02

**Authors:** Li Li, Lu Yu, Huan He, Li Wei, Zigang Xu, Libo Zhao, Yujuan Sun, Bin Zhang, Yuanxiang Liu, Rui He, Xiaoling Wang, Lin Ma

**Affiliations:** ^1^Department of Dermatology, National Center for Children's Health, Beijing Children's Hospital, Capital Medical University, Beijing, China; ^2^Clinical Research Center, National Center for Children's Health, Beijing Children's Hospital, Capital Medical University, Beijing, China; ^3^Department of Pharmacy, Peking University Third Hospital, Beijing, China

**Keywords:** infantile hemangioma, propranolol, plasma drug concentration, β1-AR, CYP2D6, efficacy, adverse reactions

## Abstract

**Background and Purpose:**

This study was conducted to explore the plasma drug concentration of propranolol in Chinese Han patients with infantile haemangioma (IH) and the influencing factors, as well as the relationship among plasma drug concentrations of propranolol, β1-AR mutation and CYP2D6 188C>T, efficacy, and safety.

**Experimental Approach:**

From January 2018 to April 2019, 140 patients with IH who were admitted to the hospital for oral propranolol and agreed to have their plasma concentration of propranolol tested, including 112 patients with β1-AR and CYP2D6 gene tested.

**Key Results and Conclusions and Implications:**

The mean peak blood levels of propranolol, 4-hydroxypropranolol (4-OH-P), and N-deisopropylpropranolol (NDP) were 60.35 ± 37.90, 1.90 ± 2.37, and 0.24 ± 0.18 ng/ml, respectively. The mean trough blood levels of propranolol, 4-OH-P, and NDP were 24.98 ± 17.68, 0.45 ± 0.52, and 0.05±0.05 ng/ml, respectively. The higher the dose of propranolol, the higher the plasma concentration of propranolol (*p* = 0.031). The plasma concentration of propranolol was not related to the treatment efficacy.

## Introduction

Infantile haemangioma (IH) is a common benign tumor in children with an incidence of 4–5% (1). Approximately 15% of IHs require treatment because the proliferation of lesion causes functional problems, such as visual or airway obstruction and ulceration or tissue distortion (2). Propranolol (non-selective beta-adrenergic receptor blocker) is effective and relatively safe in treating IH as first-line treatment (3). The main adverse reactions include hypoglycaemia, hyperkalaemia, bradycardia, hypotension, atrioventricular (AV) block, and airway hyperresponsiveness (4). The effect and safety of propranolol on IH is variable in different children, and individual differences were obvious after the oral administration of propranolol (5). The beta-blocking effects of propranolol are related to its plasma concentrations (6). It is uncertain whether the efficacy and safety of propranolol on IH are related to its plasma concentration (7, 8).

Propranolol is metabolized *in vivo* by CYP2D6 as the main metabolite 4-hydroxypropranolol (4-OH-P) and *in vivo* by CYP1A2 as N-deisopropylpropranolol (NDP) (9). Oral propranolol is mostly metabolized *in vivo* by the liver enzyme CYP2D6 (10). The correlation between the efficacy and safety of propranolol and the plasma concentrations of propranolol and its metabolite is uncertain. Meanwhile, the mechanism of propranolol for IH treatment is unclear. Adrenergic receptors include β1(β1-AR), β2 (β2-AR), and β3 (β3-AR), where β1-AR is the major adrenergic receptor in the heart (11) and in the proliferative and subsiding period of IH (12). The mean level of β1-AR mRNA expression of proliferative IH was higher than that of propranolol-treated IH (13). The correlation between CYP2D6 polymorphism and β1-AR gene mutation and the serum concentration of propranolol in the treatment of IH is unclear.

In this study, we aimed to evaluate the plasma concentration of propranolol in IH patients and analyse the relationship between the plasma concentrations of propranolol and its metabolite. Moreover, the genetic study of CYP2D6 188C>T and β1-AR was conducted to provide evidence for individualized treatment in the clinic.

## Methods

### Subject

A total of 140 patients, who were admitted for oral propranolol treatment from January 2018 to April 2019 in our center, were retrospectively analyzed. Inclusive criteria were as follows: ① Erythema or subcutaneous mass at birth or a few days after birth, with a history of rapid growth, clinical manifestations combined with an ultrasound diagnosed of IH; ② older than 1 month; ③ treated with propranolol, no concurrent use of other drugs; and ④ normal liver and kidney functions. Exclusion criteria were as follows: sinus bradycardia, heart block (II–III AV block), severe or acute heart failure, cardiogenic shock, asthma, hypothyroidism, and Raynaud's syndrome or other peripheral vascular diseases.

### Drug Administration

All patients were given oral propranolol hydrochloride. The first two doses were 0.75–1 mg/(kg d) q12h. After the first two doses, the dose was increased to 1.5–2 mg/(kg d) q12h if the heart rate, blood pressure, and blood glucose were within the normal range. Next, the dosage was maintained at 1.5–2 mg/(kg d). If the above indexes were not in the normal range, then the dosage was not increased to the maintenance dose until after 1 month of propranolol treatment.

### Informed Consent

According to the Helsinki Declaration, written informed consent was obtained from the families of participants in consideration of the protection of their rights. In addition, we explained that blood samples would not be used for purposes other than drug blood concentration testing or β1-AR and CYP2D6 gene testing. Prior to this study, the protocol was approved by the Ethics Review Board in our hospital.

### Determination of Plasma Concentration

The drug concentration in the blood was measured before the 5th dose of propranolol (trough concentration) or after taking propranolol for five times (peak concentration). If the dosage and interval were fixed, then the drug was given with a half-life interval. After 4–5 half-lives, a stable blood concentration was achieved. To understand the relationship between propranolol and its metabolite, we collected the peak and trough concentrations. The drug concentrations (propranolol, 4-OH-P, and NDP) in plasma were determined by the high-performance liquid chromatography-tandem mass spectrometry (HPLC-MS/MS) method that we established (9). The peak concentration was collected 1 h after the 5th dose, and the trough concentration was collected 1 h before the 5th dose.

### Detection of β1-AR and CYP2D6 188C>T Mutation

After extracting DNA from whole blood, primers were designed by Primer Z software, PCR amplification was carried out according to primer conditions, and sequencing was carried out by ABI 3730 first-generation sequencer.

### Observation Index and Criteria for Evaluation of Curative Effect and Side Effects

The efficacy of IH treatment was evaluated with a 4-grade scoring method as follows: Grade I: the tumor shrank by 0–25% and its surface became lighter than before; grade II: the tumor shrank by 26–50% and its color became significantly lighter than before; grade III: the tumor shrank by 51–75% and its color became significantly lighter than before; grade IV: Reduction of more than 75% or disappearance of surface color. The blood glucose level and hyperkalaemia were recorded after 1 month of propranolol treatment. Use concentration/ dose ratio (plasma concentration to each dose per body weight) to analyse the relationship among plasma concentration, efficacy and side effects.

### Statistical Methods

SPSS22.0 statistical software was used to analyse the data. Pearson correlation analysis was used for the correlation analysis. The variables that did not conform to the normal distribution were analyzed by one-way ANOVA, and the correlation of two categorical variables was tested with Fisher's precision test. The distribution of CYP2D6 188C>T genotypes in the population was examined by the Hardy–Weinberg equilibrium, and the results were verified by a chi-square test. The difference was statistically significant with *p* < 0.05.

## Results

### Patient Characteristics

A total of 140 patients, including 40 boys (28.6%) and 100 girls (71.4%) aged 30 days to 14 months, of which 126 (90.0%) were ≤6 months old and 14 (10.0%) were >6 months old. Among these, 52 were delivered by labor (37.1%) and 88 were delivered by cesarean section (62.9%). There were 17 preterm births (12.1%), 123 full-term births (87.9%), and 7 twins (5.0%). In 9 patients (6.4%), the dosage was not increased to the maintenance dose due to the decrease in heart rate and blood pressure.

### β1-AR Mutation and CYP2D6 188C>T Mutation

A total of 112 cases of β1-AR and CYP2D6 gene mutation were detected. Four cases (3.6%) of β1-AR had insertion mutation (c.837_838insTCACCC) which is led to the interinsertion of Serpro to 279_280, (p.279_280insSerPro). Two of the cases were twins. CYP2D6 188C>T genotype analysis showed that 39 patients (34.8%) were homozygous for C/C188, 46 patients (41.1%) were C/T heterozygote, and 27 patients (24.1%) were T/T188 homozygote. The allelic frequencies of CYP2D6 (188C>T) were consistent with the Hardy–Weinberg equilibrium.

### Analysis of Blood Concentration

**Figure 1:**
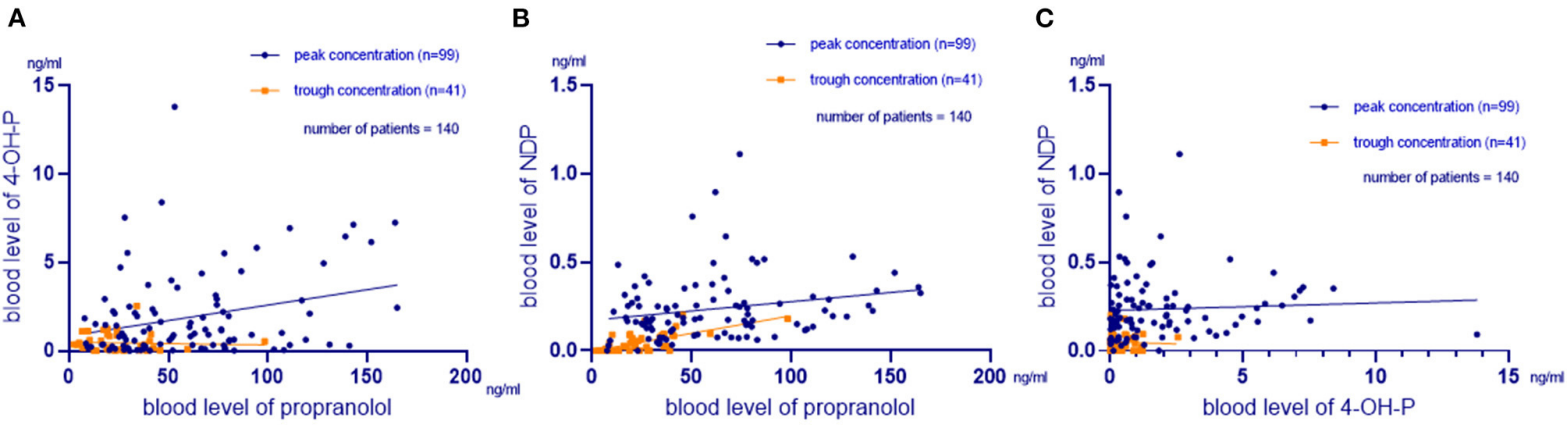
Relationship between propranolol, 4-OH-P, and NDP. **(A)** Relationship between the blood propranolol and 4-hydroxypropranolol (4-OH-P) levels. The mean blood levels of propranolol and 4-OH-P were 49.99 ± 36.93 and 1.48 ± 2.12 ng/ml, respectively. A correlation was found between the two parameters (*R*^2^ = 0.360, *p* = 0.000). **(B)** Relationship between the blood propranolol and N-deisopropylpropranolol (NDP) levels. The mean blood levels of propranolol and NDP were 49.99 ± 36.93 and 0.18 ± 0.18 ng/ml, respectively. A correlation was found between the two parameters (*R*^2^ = 0.399, *p* = 0.000). **(C)** Relationship between the blood 4-OH-P and NDP levels. The mean blood levels of 4-OH-P and NDP were 1.48 ± 2.12 and 0.18 ± 0.18 ng/ml, respectively. A correlation was found between the two parameters (*R*^2^ = 0.194, *p* = 0.021).

Peak concentration data were collected from the first 99 patients, and trough concentration data were collected from the following 41 patients. The mean peak blood levels of propranolol, 4-OH-P, and NDP were 60.35 ± 37.90 (7.67–165.00), 1.90 ± 2.37 (0.01–13.80), and 0.24 ± 0.18 (0.00–1.11) ng/ml, respectively. The mean trough blood levels of propranolol, 4-OH-P, and NDP were 24.98 ± 17.68 (2.23–98.4), 0.45 ± 0.52 (0.00–2.45), and 0.05 ± 0.05 (0.00–0.20) ng/ml, respectively. There was a correlation between the propranolol and 4-OH-P (*R*^2^ = 0.360, *p* = 0.000) (Figure 1A), between propranolol and NDP (*R*^2^ = 0.399, *p* = 0.000) (Figure 1B), and between 4-OH-P and NDP (*R*^2^ = 0.194, *p* = 0.021) (Figure 1C). In the peak concentration group, gender and preterm and twin incidences did not affect the propranolol, 4-OH-P, and NDP plasma drug concentrations. In patients who were ≤6 months old, NDP plasma drug concentration was lower than that of those aged >6 months (*F* = 42.772, *p* = 0.000). The propranolol plasma drug concentration was different in different doses. When the dose increased, the propranolol drug concentration increased as well (*F* = 3.072, *p* = 0.031). The blood concentration of the 0.75–1 mg/(kg·d) group was 29.25 ± 17.21 ng/ml, which was lower than that of the 1.5–2 mg/(kg·d) group (*p* = 0.009). No significant difference was found between 0.75–1 and 1.5–2 mg/(kg·d) groups in 4-OH-P and NDP (*p* = 0.926 and *p* = 0.403, respectively). In the mutation group of β1-AR, the plasma concentration of 4-OH-P was higher than in the normal group of β1-AR (*p* = 0.001). In C/C 188 homozygous of CYP2D6, the plasma drug concentration of propranolol was lower than in T/T 188 homozygous, but the difference was not significant (*p* = 0.063). The plasma drug concentration of NDP of C/C 188 was lower than that of T/T 188 homozygous (*p* = 0.014), as shown in Table 1.

**Table 1:** The factors affecting the drug blood concentration in the peak concentration group.

	**Propranolol (ng/ml)**	***F***	***P***	**4-OH-P (ng/ml)**	***F***	***P***	**NDP (ng/ml)**	***F***	***P***
**Gender**
Male (31)	63.84 ± 45.11	0.382	0.538	2.28 ± 2.47	1.146	0.287	0.27 ± 0.22	1.715	0.193
Female (68)	58.75 ± 34.38			1.73 ± 2.33			0.22 ± 0.16		
**Age**
≤6 months (91)	59.86 ± 38.09	0.162	0.688	1.84 ± 2.33	0.528	0.469	0.20 ± 0.13	42.772	0.000^**^
>6 months (8)	65.22 ± 37.83			2.45 ± 2.91			0.55 ± 0.28		
**Preterm**
Yes (8)	75.83 ± 47.31	1.461	0.230	1.31 ± 1.04	0.528	0.469	0.30 ± 0.21	1.058	0.306
No (91)	58.98 ± 36.98			1.95 ± 2.45			0.23 ± 0.18		
Twins									
Yes (7)	59.23 ± 35.68	0.006	0.936	3.12 ± 2.63	2.021	0.158	0.29 ± 0.13	0.605	0.438
No (92)	60.43 ± 38.25			1.81 ± 2.34			0.23 ± 0.19		
**Dose (mg/kg d)**
0.75 (7)	31.18 ± 19.12	3.072	0.031^**^	1.40 ± 1.68	2.707	0.050	0.19 ± 0.14	1.784	0.155
1 (2)	22.5 ± 7.64			3.96 ± 5.07			0.18 ± 0.01		
1.5 (48)	58.28 ± 35.96			1.32 ± 1.50			0.20 ± 0.14		
2 (42)	69.37 ± 39.91			2.54 ± 2.96			0.28 ± 0.22		
**β1-AR**
Normal (95)	61.49 ± 38.24	2.147	0.146	1.74 ± 2.23	11.862	0.001^**^	0.24 ± 0.18	0.004	0.951
Mutation (4)	33.30 ± 9.33			5.70 ± 2.85			0.23 ± 0.09		
**CYP2D6 188C>T**
CC (35)	51.58 ± 35.01	2.849	0.063	2.33 ± 2.87	1.475	0.234	0.20 ± 0.20	4.426	0.014^**^
CT (39)	59.03 ± 37.17			1.92 ± 2.29			0.21 ± 0.12		
TT (25)	74.67 ± 40.17			1.27 ± 1.53			0.33 ± 0.21		

*CC, C/C 188 homozygous of CYP2D6; CT, C/T 188 heterozygous of CYP2D6; TT, T/T 188 homozygous of CYP2D6*;

* *p < 0.05*;

** *p < 0.01*.

In the trough concentration group, gender, age, preterm, and dose did not affect the propranolol, 4-OH-P, and NDP plasma drug concentrations. No twins, no side effects, and no β1-AR mutation occurred in the trough concentration group at initial treatment. In the C/C 188 homozygous of CYP2D6, the plasma drug concentration of propranolol was lower than that in the T/T 188 homozygous (*p* = 0.042), as shown in Table 2.

**Table 2:** The factors affecting the drug blood concentration in the trough concentration group.

	**Propranolol (ng/ml)**	***F***	***P***	**4-OH-P (ng/ml)**	***F***	***P***	**NDP (ng/ml)**	***F***	***P***
**Gender**
Male (9)	19.51 ± 12.67	1.106	0.299	0.51 ± 0.37	0.123	0.728	0.03 ± 0.05	0.830	0.368
Female (32)	26.52 ± 18.72			0.44 ± 0.56			0.05 ± 0.06		
**Age**
≤6 months (36)	25.81 ± 18.30	0.654	0.423	0.47 ± 0.53	0.239	0.627	0.05 ± 0.05	0.637	0.430
>6 months (5)	18.96 ± 11.99			0.35 ± 0.47			0.07 ± 0.06		
**Preterm**
Yes (9)	23.77 ± 14.64	0.052	0.820	0.49 ± 0.44	0.048	0.828	0.06 ± 0.07	0.201	0.656
No (32)	25.32 ± 18.66			0.44 ± 0.54			0.05 ± 0.05		
**Dose (mg/kg d)**
1.5 (24)	24.50 ± 20.01	0.041	0.840	0.40 ± 0.36	0.615	0.438	0.04 ± 0.05	1.286	0.264
2 (17)	25.65 ± 14.30			0.53 ± 0.69			0.06 ± 0.06		
**CYP2D6**
CC (4)	10.23 ± 9.68	4.421	0.042^*^	0.62 ± 0.40	0.141	0.870	0.00 ± 0.00	1.282	0.319
CT (7)	20.87 ± 13.02			0.50 ± 0.46			0.03 ± 0.05		
TT (2)	39.60 ± 2.26			0.66 ± 0.41			0.05 ± 0.04		

*CC, C/C 188 homozygous of CYP2D6; CT, C/T 188 heterozygous of CYP2D6; TT, T/T 188 homozygous of CYP2D6*;

* *p < 0.05*.

### Relationship Between Plasma Drug Concentration, Efficacy, and Safety

Nine cases in the peak concentration group were observed to show bradycardia at the initial five doses. Among these nine cases, seven cases were given 0.75 mg/(kg·d) and two cases were given 1 mg/ (kg·d). No patient received a dose increase due to decreased heart rate, blood pressure, or blood glucose in the trough concentration group.

After oral propranolol treatment at 1.5 or 2 mg/(kg·d) for 1 month, 84 patients (84.8%) with peak concentration and 37 patients (90.2%) with trough concentration were followed up. No relationship was found between the concentration/ dose ratio of propranolol (peak concentration), 4-OH-P (peak concentration) and NDP (peak concentration) and efficacy (*p* = 0.663, *p* = 0.087, and *p* = 0.110, respectively). There was no relationship between concentration/ dose ratio of propranolol (trough concentration), 4-OH-P (trough concentration), and NDP (trough concentration) and efficacy (*p* = 0.324, *p* = 0.155, and *p* = 0.964, respectively).

One year after oral propranolol treatment at 1.5 or 2 mg/(kg·d), efficacy information was collected from 62 patients (62.6%) with peak concentration and 32 patients (78.0%) with trough concentration. There was no significant difference in the concentrations of propranolol and 4-OH-P between the peak and trough concentration groups after 1 year.

Among 84 patients with peak concentration, 75 patients developed normal serum potassium and 9 patients developed hyperkalaemia. No relationship was found between the concentration/ dose ratio of propranolol, 4-OH-P (peak concentration), and NDP (peak concentration) and hyperkalaemia (*p* = 0.691, *p* = 0.687, and *p* = 0.270, respectively). Among 37 patients with trough concentration, 36 patients developed normal serum potassium and 1 patient developed hyperkalaemia.

Among 84 patients with peak concentration, 82 patients developed normal blood glucose and 2 patients developed hypoglycaemia. No relationship was found between concentration/ dose ratio of propranolol, 4-OH-P (peak concentration), and NDP (peak concentration) and hypoglycaemia (*p* = 0.771, *p* = 0.242, and *p* = 0.482, respectively). In 37 patients with trough concentration, the blood glucose was normal.

### Relationship Between β1-AR and CYP2D6 188C>T and Adverse Reaction

During the initial part of the treatment, four patients subjected to mutation tests did not receive an increased dose due to bradycardia. Patients with β1-AR gene mutation was more likely to show bradycardia (*p* = 0.032). There was no significant correlation between CYP2D6 188C>T and bradycardia during the first five times of administration. After 1 month of treatment, the occurrence of hyperkalaemia and hypoglycaemia were not related to β1-AR mutation or CYP2D6 188C>T (as shown in Table 3).

**Table 3:** β1-AR, CYP2D6 gene mutation and adverse reactions.

	**Normal heart rate**	**Bradycardia**	***P***	**Normal potassium**	**Hyperkalaemia**	***P***	**Normal blood glucose**	**Hypoglycemia**	***P***
β1-AR			0.032^*^			0.290			0.942
Normal	101	7		90	10		98	2	
Mutation	2	2		2	1		3	0	
CYP2D6			0.355			0.391			0.743
CC	34	5		36	2		37	1	
CT	44	2		36	6		41	1	
TT	25	2		20	3		23	0	

*CC, C/C 188 homozygous of CYP2D6; CT, C/T 188 heterozygous of CYP2D6; TT, T/T 188 homozygous of CYP2D6*;

* *P < 0.05*.

## Discussion

Plasma propranolol concentrations varied more widely after single oral doses (seven-fold) than after intravenous doses (two-fold) in the same young volunteers (14). The plasma concentration also varied widely in IH patients with 1.5 or 2 mg/(kg·d), i.e., 21.5-fold to 44.1-fold in propranolol after 4–5 doses in different individuals. Chinese Han patients may be more sensitive to propranolol than white patients. This might be related to the wider fluctuation and individual differences of propranolol plasma concentration.

In patients aged ≤6 months, NDP plasma drug concentration (peak concentration) was lower than in those aged >6 months. This might result from the fact that the CYP1A2 value does not reach the adult value. CYP1A2 was absent in fetal and neonatal microsomal livers. Samples that attained 10–15% of the adult values in one-month-old to three-month-old infants reached 20–25% of adult values in three-month-old to one-year-old infants (15). The propranolol plasma concentration of 0.75–1 mg/(kg·d) group was lower than that of 1.5–2 mg/(kg·d) group. In the mutation group of β1-AR, the plasma concentration of 4-OH-P was higher than in the normal group of β1-AR. Polymorphism of β1-AR is associated with the cardiovascular response to metoprolol (16). At present, the relationship between β1-AR gene mutation and propranolol plasma concentration or clinical efficacy has not been proposed, and further research is needed to confirm this. The plasma trough concentration of propranolol was lower in C/C 188 homozygous of CYP2D6 than in T/T 188 homozygous. The plasma peak concentration of NDP of C/C 188 was lower in C/C 188 homozygous of CYP2D6 than in T/T 188 homozygous. The plasma concentration of propranolol varied in CYP2D6 188C>T, in accordance with a previous study (17). Whether liver blood flow, smoking difference, racial difference, and dietary difference might affect the blood concentration of propranolol was not specified (5).

In this study, the peak and trough concentrations of propranolol, 4-OH-P, and NDP were measured at 1.5, 2, 0.75, and 1 mg/kg. The concentration/ dose ratio of propranolol and its' metabolites cannot predict the efficacy in 1 month and 1 year after treatment. Further research is needed on the factors that cause individual differences in efficacy.

The plasma concentrations of propranolol and its metabolite were not correlated with hyperkalaemia and hypoglycaemia after 1 month of treatment. The β1-AR mutation might be related to bradycardia at initial treatment. However, comparisons related with β1-AR are not reliable because only 4 patients had mutations. The β1-AR mutation and CYP2D6 188C>T were not correlated with hyperkalaemia and hypoglycaemia after 1 month of treatment. The correlation between side effects at initial treatment and plasma concentration was not analyzed due to plasma concentration was tested after the occurrence of side effects at initial treatment with dose adjustment.

This is a retrospective study, and therefore, the selection bias cannot be ignored. If the drug is given with a half-life interval, then a stable blood concentration is achieved after 4–5 half-lives. The half-life of propranolol clearance *in vivo* is 2–3 h, but propranolol was administered at a fixed interval of 12 h. More pharmacokinetics research is needed to determine whether the blood concentration measured is stable *in vivo*. The mechanism of propranolol for IH treatment has not been fully elucidated; possible mechanisms include vasoconstriction, inhibition of angiogenesis, and induction of apoptosis (18). Meanwhile, the CYP2D6 gene is highly genetically polymorphic with numerous CYP2D6 allelic variants (10, 19), and their polymorphism needs further research. Propranolol is metabolized not only by CYP2D6 but also by CYP3A4 and CYP1A2. Compared with β1-AR, β2-AR might play a more important role in the treatment of IH (20, 21). β1-AR is not the only major adrenergic receptor in the heart and cardiovascular system, and so the polymorphism of β1-AR could be related to the side effects of propranolol, but not the efficacy. The gene mutations of β2-AR, CYP3A4, and CYP1A2 were not studied.

## Conclusion

The higher the dose of propranolol was, the higher the plasma concentration of propranolol. There was no significant correlation between the efficacy of propranolol in the treatment of infantile haemangioma and propranolol concentration/ dose ratio, the factors that cause individual differences in efficacy need further study.

## Data Availability Statement

The data that support the findings of this study are available from the corresponding author upon reasonable request. Some data may not be made available because of privacy or ethical restrictions.

## Ethics Statement

The studies involving human participants were reviewed and approved by Medical Ethics Committee of Beijing Children's Hospital, Capital Medical University. Written informed consent to participate in this study was provided by the participants' legal guardian/next of kin.

## Author Contributions

LM and XW: conceptualized, designed the study, and reviewed and revised the manuscript. LL and LY: conceptualized and designed the study, drafted the initial manuscript, designed the data collection instruments, collected data, carried out the initial analyses, reviewed and revised the manuscript, and critically reviewed the manuscript for important intellectual content. HH, LW, ZX, LZ, YS, BZ, YL, and RH: collected data, contributed to analysis and interpretation of data, coordinated, supervised data collection and critically reviewed it for important intellectual content. All authors contributed to the article and approved the submitted version.

## Funding

This work was supported by Capital Health Research and Development of Special (2016-2-2093); Beijing Municipal Administration of Hospitals Incubating Program (PX2016014); The Special Fund of the Pediatric Medical Coordinated Development Center of Beijing Hospitals Authority (No. XTZD20180502).

## Conflict of Interest

The authors declare that the research was conducted in the absence of any commercial or financial relationships that could be construed as a potential conflict of interest.

## Publisher's Note

All claims expressed in this article are solely those of the authors and do not necessarily represent those of their affiliated organizations, or those of the publisher, the editors and the reviewers. Any product that may be evaluated in this article, or claim that may be made by its manufacturer, is not guaranteed or endorsed by the publisher.
